# Catecholaminergic polymorphic ventricular tachycardia patients with multiple genetic variants in the PACES CPVT Registry

**DOI:** 10.1371/journal.pone.0205925

**Published:** 2018-11-07

**Authors:** Thomas M. Roston, Omid Haji-Ghassemi, Martin J. LaPage, Anjan S. Batra, Yaniv Bar-Cohen, Chris Anderson, Yung R. Lau, Kathleen Maginot, Roman A. Gebauer, Susan P. Etheridge, James E. Potts, Filip Van Petegem, Shubhayan Sanatani

**Affiliations:** 1 Departments of Medicine, Pediatrics, and Biochemistry & Molecular Biology, University of British Columbia, Vancouver, BC, Canada; 2 Department of Medicine, University of Alberta, Edmonton, AB, Canada; 3 Department of Pediatrics, University of Michigan, Ann Arbor, MI, United States of America; 4 Department of Pediatrics, University of California at Irvine Medical Center, Irvine, CA, United States of America; 5 Department of Pediatrics, Children’s Hospital Los Angeles, Los Angeles, CA, United States of America; 6 Providence Sacred Heart Children’s Hospital, Spokane, WA, United States of America; 7 Division of Pediatric Cardiology, University of Alabama at Birmingham, Birmingham, AB, United States of America; 8 Department of Pediatrics, University of Wisconsin School of Medicine & Public Health, Madison, WI, United States of America; 9 Department of Pediatric Cardiology, University of Leipzig, Leipzig, Germany; 10 Department of Pediatrics, University of Utah, and Primary Children’s Hospital, Salt Lake City, UT, United States of America; Pennsylvania State University, UNITED STATES

## Abstract

**Background:**

Catecholaminergic polymorphic ventricular tachycardia (CPVT) is often a life-threatening arrhythmia disorder with variable penetrance and expressivity. Little is known about the incidence or outcomes of CPVT patients with ≥2 variants.

**Methods:**

The phenotypes, genotypes and outcomes of patients in the Pediatric and Congenital Electrophysiology Society CPVT Registry with ≥2 variants in genes linked to CPVT were ascertained. The American College of Medical Genetics & Genomics (ACMG) criteria and structural mapping were used to predict the pathogenicity of variants (3D model of pig RyR2 in open-state).

**Results:**

Among 237 CPVT subjects, 193 (81%) had genetic testing. Fifteen patients (8%) with a median age of 9 years (IQR 5–12) had ≥2 variants. Sudden cardiac arrest occurred in 11 children (73%), although none died during a median follow-up of 4.3 years (IQR 2.5–6.1). Thirteen patients (80%) had at least two *RYR2* variants, while the remaining two patients had *RYR2* variants plus variants in other CPVT-linked genes. Among all variants identified, re-classification of the commercial laboratory interpretation using ACMG criteria led to the upgrade from variant of unknown significance (VUS) to pathogenic/likely pathogenic (P/LP) for 5 variants, and downgrade from P/LP to VUS for 6 variants. For *RYR2* variants, 3D mapping using the RyR2 model suggested that 2 VUS by ACMG criteria were P/LP, while 2 variants were downgraded to likely benign.

**Conclusions:**

This severely affected cohort demonstrates that a minority of CPVT cases are related to ≥2 variants, which may have implications on family-based genetic counselling. While multi-variant CPVT patients were at high-risk for sudden cardiac arrest, there are insufficient data to conclude that this genetic phenomenon has prognostic implications at present. Further research is needed to determine the significance and generalizability of this observation. This study also shows that a rigorous approach to variant re-classification using the ACMG criteria and 3D mapping is important in reaching an accurate diagnosis, especially in the multi-variant population.

## Introduction

Catecholaminergic polymorphic ventricular tachycardia (CPVT) is a rare inherited arrhythmia syndrome characterized by ventricular tachycardia (VT) provoked by adrenergic stress. [1] The condition is caused by excessive calcium leak from the sarcoplasmic reticulum, leading to delayed after-depolarizations and arrhythmias. [1] Most cases are attributed to mutations in *RYR2-*coded ryanodine receptor (RyR2) or *CASQ2-*coded calsequestrin-2 [1]. Although less recognized, *SCN5A*, *TRDN*, and *CALM1-3* have also been implicated in catecholamine sensitive polymorphic VT. [2–7]

To date, genotype-based risk predictors have not been clinically useful. In other inherited arrhythmic conditions, like long QT syndrome (LQTS) and hypertrophic and arrhythmogenic cardiomyopathies, patients with double and compound mutations fare especially poorly. [8–12] We used the Pediatric and Congenital Electrophysiology Society (PACES) Registry [13, 14] to characterize CPVT patients with ≥2 variants. To systematically assess the likelihood of pathogenicity, variants were mapped on to the 3D structure of RyR2, which provides mechanistic insights into their function and enhances the analysis compared to sequence-based scoring algorithms alone.

## Material and methods

**Fig 1:**
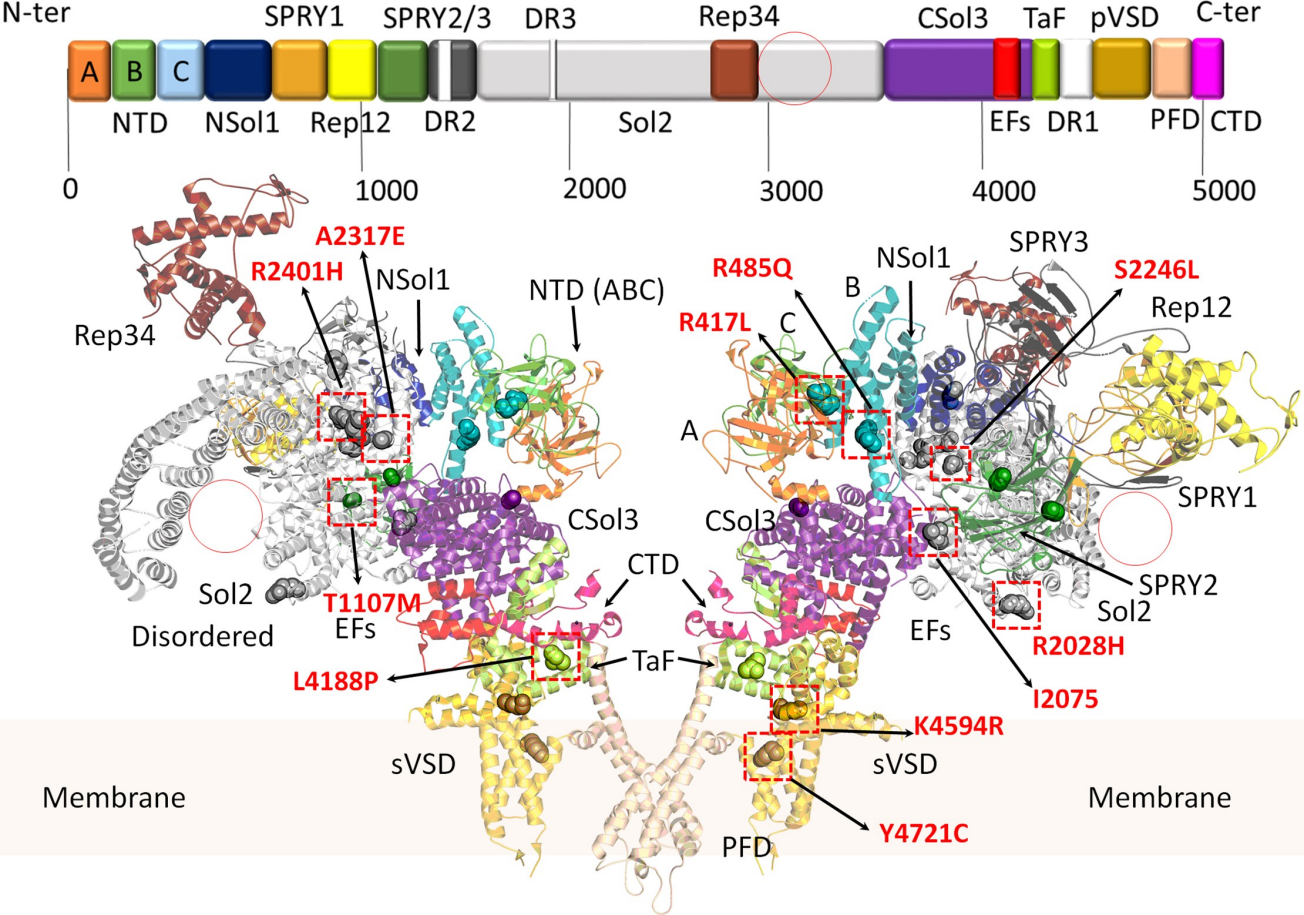
Domain architecture of the cardiac RyR2. Domains are coloured according to the linear sequence scheme shown above the ribbon diagram. Structure obtained from PDB: 5GOA. [15] The ribbon diagram shows a RyR2 dimer from the side. Red circles represent large alpha solenoid regions which are unstructured in the cryo-electron micrograph (CryoEM) structure. All variants discussed in the manuscript are shown in sphere representations. Red squares highlight location of the most likely damaging variants (expanded in Fig 2). Domains are named according to nomenclature used by des Georges, et al., 2016. A (*dark orange*), B (*green*), and C (*light blue*) domains: form part of the N-terminal domain (NTD); NSol (*dark blue*): alpha-solenoid region near the NTD; SPRY1 (*light orange*), SPRY2 (*dark green*), and SPRY3 (*dark grey*): three domains named after splA kinase and RyRs where they were first identified; Sol2 (*light grey*): second alpha-solenoid region centrally located on RyR2; Rep12 (*yellow*) and Rep23 (*brown*): four repeats (~100 aa each) in two tandem arrangements, Repeats 1 and 2 located between SPRY 1 and SPRY2, and Repeats 3 and 4 located within Sol2; CSol3 (*magenta*): third alpha-solenoid region located near the C-terminal; EFs (*red*): pair of EF hand-like motifs located within CSol3 region; TaF (*light green*): thumb and forefingers domain; DR1/2/3 (*white*): evolutionary divergent regions of RyR isoforms (not shown in the ribbon diagrams); pVSD (*gold*): pseudo voltage-sensing domain; PFD (*wheat*): pore-forming domain; CTD (*pink*): C-terminal domain.

This is a retrospective study derived from the PACES CPVT Registry, which is an international multicenter registry of children (≤19 years) and their first-degree relatives with a diagnosis of CPVT made by consensus criteria. [1] Clinical, genotypic and outcome data were previously reported. [13, 14] Participating sites received ethical approval locally and the protocol adhered to the 1975 Declaration of Helsinki. The coordinating center was responsible for data collection and analysis, and the protocol was approved by the UBC C&W Research Ethics Board. The review board did not require individual patient consent as the study was a retrospective chart review. All data were de-identified prior to entry and analysis. A stepwise bioinformatics approach was implemented to classify variant pathogenicity, including structural mapping using 3D model of pig RyR2 in open-state (Fig 1). Continuous data are presented as the median (interquartile range). Detailed methods are available in the supporting supplemental information (S1 File and S2 File).

## Results

### Population

Of 237 patients entered in the PACES CPVT Registry, 193 (81%) underwent genetic testing. There were 15 patients (8%) from 12 families with ≥2 variants. Table 1 summarizes the genotypes and phenotypes of these multi-variant carriers. The median age at presentation was 9 years (IQR 5–12) and 9 (60%) were female. Thirteen children (87%) had ≥2 *RYR2* variants, one had *CASQ2* and *RYR2* variants, and one had *RYR2* and *SCN5A* variants (Table 1, Table 2 and S2 File). There were 12 children (80%) who were probands. Inheritance could not be established in 6 children (40%) owing to a lack of parental genetic data. A family history of suspected/confirmed CPVT was reported in 10 patients (67%). VT and/or sudden cardiac arrest (SCA) occurred in 13 of 15 cases (87%). The exceptions were subject #6 who was asymptomatic and subject #12 who had exertional syncope and seizures. Pedigrees for patients from select families are available in the supporting information (S3 File).

**Table 1:** Clinical characteristics and outcomes of multi-variant carriers.

Subject	Sex	Ethnicity	Age (years)	Proband	Variant #1	Variant #2	Inheritance	Phase (cis vs trans)	Family History	Symptoms	Treatments	Treatment Failure
1	F	Hispanic	16	Yes	*RYR2*-p.R417L	*RYR2*-p.F3496L	Paternal	Cis	Father is gene carrier, asymptomatic	Exertional syncope, exertional VT	Nadolol	No
2	F	Hispanic	9	No	*RYR2*-p.R417L	*RYR2*-p.F3496L	Paternal	Cis	Sibling of subject 1	Family screening, exertional VT	Untreated	N/A
3	M	Caucasian	12	Yes	*RYR2-*p.S3938R	*RYR2-*p.R485Q	Unknown	Unknown	No suspected/known CPVT	Exertional SCA	Atenolol & ICD	No
4	F	Caucasian	12	Yes	*RYR2-*p.I2075T	*RYR2-*p.K4594R	Obligate paternal inheritance	Cis	Positive for SCA in sister	SCA	Metoprolol & ICD	No
5	F	Caucasian	10	No	*RYR2-*p.I2075T	*RYR2-*p.K4594R	Obligate paternal inheritance	Cis	Sister of subject 5	Exertional SCA	Metoprolol & ICD	No
6	F	Caucasian	5	No	*RYR2-*p.I2075T	*RYR2-*p.K4594R	Paternal inheritance	Cis	Paternal cousin of subject 5	Asymptomatic	ICD	N/A
7	F	Caucasian	12	Yes	*RYR2-*p.R2028H	*RYR2-*p.Y4721C	Variant #1 from mother, variant #2 from father	Trans	Parents are phenotypically silent heterozygous carriers	Exertional SCA	Atenolol & ICD	Yes
8	M	Arab	7	Yes	*RYR2-*p.T1107M	*CASQ2-*c.IVS5+1G>C (homozygous)	*CASQ2* inherited from consanguineous parents	Trans	Parents are first cousins No suspected/known CPVT	Exertional syncope, SCA, VT on EST	Nadolol, flecainide, ICD, sympathectomy	Yes
9	M	Caucasian	newborn	Yes	*RYR2-*p.R2474K	*RYR2-*p.A1136V	De novo	Unknown	No suspected/known CPVT	Exertional SCA	Atenolol, later changed to nadolol & ICD	Yes
10	F	Hispanic	11	Yes	*RYR2-*p.L4188P	*RYR2-*p.G1886S	Unknown	Unknown	No suspected/known CPVT	Seizures, emotional SCA	Nadolol	No
11	F	Caucasian	4	Yes	*RYR2-*p.S2246L	*RYR2-*p.G1886S	Unknown	Unknown	No suspected/known CPVT	SCA	Nadolol & ICD	No
12	M	Caucasian	9	Yes	*RYR2-*p.H2464D	*RYR2-*p.G1885E	Mother gene negative, father unknown	Unknown	No suspected/known CPVT	Exertional syncope, epilepsy	Atenolol, later changed to nadolol, flecainide & valproate	No
13	M	White	7	Yes	*RYR2-*p.R2401H	*DSG-*p.V288I	Unknown	Unknown	No suspected/known CPVT	Exertional SCA	Nadolol	No
14	M	White	5	Yes	*RYR2-*p.G4772S	Multiple*	Unknown	Unknown	SCA in multiple relatives (symptomatic cousin carries *RYR2-*G4772S)	Exertional syncope, SCA, & VT storm	Nadolol & ICD	Yes
15	F	Arab	8	Yes	*RYR2-*p.A2317E	*SCN5A-*p.Q692K	Unknown	Unknown	Sudden death in maternal grandfather (swimming at 39 years old)	Exertional SCA	Nadolol, ICD & LCSD	No

*Subject 14 had additional variants as follows: *RYR2-*c.3599-9delT, *RYR2-*c.14091-11dupT, *CACNA1c-*p.T1870M, *CACNA1C-*c.5680+11C>T, *TMEM43-*c.512+19G>T, *PKP2-*c.2300-4G>C, *DSP-*p.R1458G.

N/A = not applicable

**Table 2:** Clinical and molecular data supporting variant classification.

Subject(s)	Variants	Reported pathogenicity from commercial testing lab	ExAC browser allele frequency	Pathogenicity re-classification based on ACMG Criteria	Predicted structural impact based on RyR2 model
1, 2	*RYR2*-p.R417L (Fig 2A)	P/LP	Absent	Likely Pathogenic	R417 is located near the anion-binding site in domain C, at domains A-C and B-C interfaces. The inter-domain area is dominated by hydrophilic and charged residues. The R417L variant would introduce a shorter, hydrophobic side chain in place of a bulky, positively charged side chain, which may alter the anion binding and cause domain-domain rearrangements.
	*RYR2*-p.F3496L	VUS	Absent	Likely Pathogenic	F3496 is located in an intrinsically disordered alpha-solenoidal region of RyR2 (Sol2).
3	*RYR2-*p.R485Q (Fig 2A)	VUS	0.00008645	Likely Pathogenic	R485 is located inside an alpha helix of domain C, buried within the helical bundle. The R485 side chain forms a salt bridge with the E411, located in another helix facing domains A and B. The R485Q variant would break this interaction, destabilizing domain C, and affect the anion binding site.
	*RYR2-*p.S3938R	P/LP	Absent	Likely Pathogenic	S3938 is located in the CSol3 region of RyR2. S3938 is near the pore, within the cytosolic side of the channel. Mutation to bulkier, positively charged side chain may alter hydrogen bonding pattern at this site and/or disrupt surrounding alpha helices structure.
4, 5, 6	*RYR2-*p.I2075T (Fig 2B)	P/LP	Absent	VUS	I2075 is located within the Sol2 region, where it is buried between two helices. The Ile residue is surrounded by hydrophobic residues. The variant is close to an interface with Csol3 region, and thus the variant may impact this inter-domain interaction.
	*RYR2-*p.K4594R (Fig 2C)	VUS	Absent	Likely Pathogenic	K4594 is located at the cytosolic edge of the pseudo voltage-sensing domain (pVSD), next to the thumb and forefingers (TaF) domain. These domains are implicated in the binding of activating ligands and channel opening. Although the K4594R substitution is conservative, the guanidinium group of Arg allows for a larger number of interactions or may facilitate a stronger interaction with nearby E4200. The ATP/Caffeine binding sites located nearby, thus any small perturbation in this area is likely to alter channel gating.
7	*RYR2-*p.R2028H	P/LP	Absent	VUS	R2028 is found in Sol2 region of RyR2, pointing toward the solvent. The variant is unlikely to have a major impact on the function, but may influence binding to an unknown auxiliary protein.
	*RYR2-*p.Y4721C (Fig 2D)	P/LP	Absent	Likely Pathogenic	This residue is located within the transmembrane region of pVSD. This region plays an important role in allosteric gating of the channel and the Tyr is surrounded by other hydrophobic residues. Mutation to cysteine is likely to perturb channel gating and domain packing.
8	*RYR2-*p. T1107M (Fig 3A)	VUS	Absent	Pathogenic	T1107 is located within the SPRY2 domain, where it is buried and surrounded by hydrophobic residues. The variant would form steric clashes with W1156 and cause destabilization of the domain, as shown in a crystallographic study of this mutant[19]. Functional experiments have shown it affects Ca^2+^ release properties (see suppl. table 1).
	*CASQ2-*c.IVS5+1G>C	P/LP	Absent	VUS	Not performed
9	*RYR2-*p.R2474K (Fig 3B)	P/LP	Absent	Likely pathogenic	R2474 is located in the Sol2 region of RyR2, near two other mutations. Region is poorly resolved in CryoEM structures. The variant is subtle and structural predicted suggests a minimal impact. It is currently unknown whether any auxiliary protein binds to this region.
	*RYR2-*p.A1136V	VUS	0.007063	Likely pathogenic	A1136 is located within the SPRY2 domain. The equivalent residue in both RyR1 and RyR3 is a valine, therefore the mutation is unlikely to have significant negative impact on the overall structure of RyR.
10	*RYR2-*p.L4188P (Fig 3C)	VUS	Absent	VUS	L4188 is located within a helix as part of the TaF domain that clamps the C-terminal extension of the RyR. This interaction is critical for channel gating. The substitution to Pro may promotes helix breaking, and potentially perturb channel gating.
	*RYR2-*p.G1886S	VUS	0.04385	VUS	G1886 is located in a flexible unstructured loop as part of Sol2 region. Though the substitution alone is unlikely to have an impact on channel gating, it may have indirect effects such as creation of a new phosphorylation site.
11	*RYR2-*p.S2246L (Fig 3D)	P/LP	Absent	Pathogenic	S2246 is located within the Sol2 region, where the side chain is tightly packed next to an alpha helix. Mutation to a longer side chain likely results in steric clashes, and will impact helix packing in this region.
	*RYR2-*p.G1886S	VUS	0.01540	VUS	G1886 is located in a flexible unstructured loop as part of Sol2 region. Though the substitution alone is unlikely to have an impact on channel gating, it may have indirect effects such as creation of a new phosphorylation site or alter bidding to auxiliary protein(s).
12	*RYR2-*p.H2464D	P/LP	Absent	Pathogenic	H2464 is located within a poorly resolved Sol2 region of RyR2 structure. The variant may impact binding of an unknown auxiliary protein to this region.
	*RYR2-*p.G1885E	VUS	0.04385	VUS	G1885 is located in a flexible unstructured loop of RyR2, and thus cannot be mapped onto the existing structure.
13	*RYR2-*p.R2401H (Fig 3B)	P/LP	Absent	Likely Pathogenic	R2401 is located within the Sol2 region, near two other CPVT associated mutations. Substitution to His may have an impact on helix stability.
	*DSG-*p.V288I	VUS	Absent	VUS	Not performed
14	*RYR2-*p.G4772S	P/LP	Absent	VUS	G4772 is located in the pore forming domain (PFD), as part of the outer helix. Substitution to less flexible Ser may affect helical packing within the membrane and cause subtle domain rearrangements.
	*CACNA1c-*p.T1870M	VUS	Absent	VUS	Not performed
	*RYR2-*c.3599-9delT	VUS	Absent	VUS	Not performed
	*RYR2-*c.14091-11dupT	VUS	Absent	VUS	Not performed
	*CACNA1C-*c.5680+11C>T	VUS	Absent	VUS	Not performed
	*TMEM43-*c.512+19G>T	VUS	Absent	VUS	Not performed
	*PKP2-*c.2300-4G>C	VUS	0.00008079	VUS	Not performed
	*DSP-*p.R1458G	P/LP	0.001737	VUS	Not performed
15	*RYR2-*p.A2317E (Fig 3B)	P/LP	Absent	VUS	A2317 is in an alpha solenoid region, near two other CPVT associated mutations. Mutation to the larger Glu residue likely forms steric clashes with nearby residues, and this is likely to affect packing and stability of the region.
	*SCN5A-*p.Q692K	P/LP	0.0002822	VUS	Mutation site is on the 1–2 linker (DI-II loop) near the start the VSD of the cardiac voltage-gated sodium channel (NaV1.5). The region is important to gating function of the channel and is in close proximity to the CaMKII binding site. The mutation may also influence binding to other auxiliary proteins.

### Therapies & outcomes

Anti-arrhythmic therapy was instituted in 13 of 15 patients (87%). One patient received no treatment (subject #2), and in one patient, only an implantable cardioverter-defibrillator (ICD) was used (subject #6). A beta-blocker was prescribed in all treated patients. Therapeutic escalation with flecainide and left cardiac sympathectomy was necessary in subject #8 due to arrhythmias on beta-blockers. He continued to have events despite these ancillary treatments. Subject #12 also received flecainide for refractory arrhythmias and valproate for seizures, while subject #15 eventually had a left cardiac sympathectomy. An ICD was implanted in 10 of 15 patients (67%), 9 of which were for secondary prevention after SCA. Treatment failure occurred in 4 of 13 subjects (31%) on medication. No patients died during a median follow-up of 4.3 years (IQR 2.5–6.1).

### Genetic analysis

**Fig 2:**
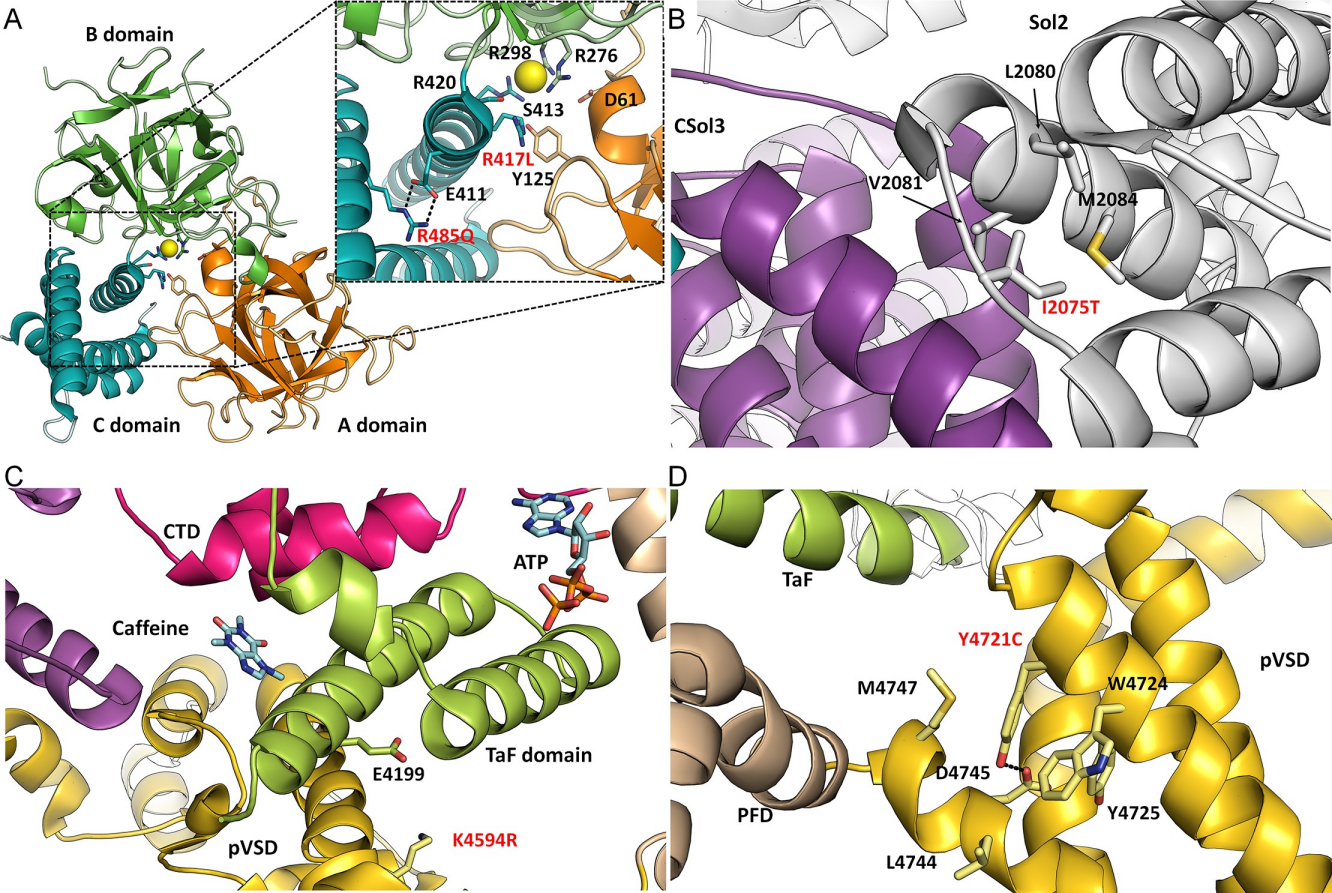
Location of CPVT-associated variants on RyR2 structure. Enlarged view of the insets are presented to highlight location of the variants and nearby residues/secondary structures. Domains are coloured according to the linear sequence scheme shown in Fig 1. All variants occur in highly conserved regions of all three RyR isoforms. Unless specified, the structures are based on the 4.2 Å resolution CryoEM open-state structure of RyR2 from porcine heart[15], PDB: 5GOA. (**A**) *RYR2-*p.R417L and *RYR2*-p.R485Q mutants are located centrally within RyR2 ABC domains, near the anion binding site. Residues involved in the formation of the Cl^-^ pocket are highlighted. R485 forms a salt bridge interaction with nearby E411 residue. (**B**) The *RYR2*-p.I2075T is located within a flexible linker, joining two alpha-helices together in the Sol2 region. The Ile is surrounded by nearby hydrophobic residues. (**C**) The *RYR2*-p.K4594R variant is located in the pVSD domain, near the TaF domain. Mutation to Arg, may potentially form a salt bridge with a nearby E4199 located on the TaF domain. In RyR1 open-state structure[20] the K4594 is located near activating ligands such as caffeine and ATP, PDB: 5TAQ. (**D**) The *RYR2*-p.Y4721C variant is located within the transmembrane region of pVSD, and unsurprisingly the Y4721 is surrounded by other hydrophobic residues. Y4721 is potentially forming a polar contact with carboxyl group D4745.

**Fig 3:**
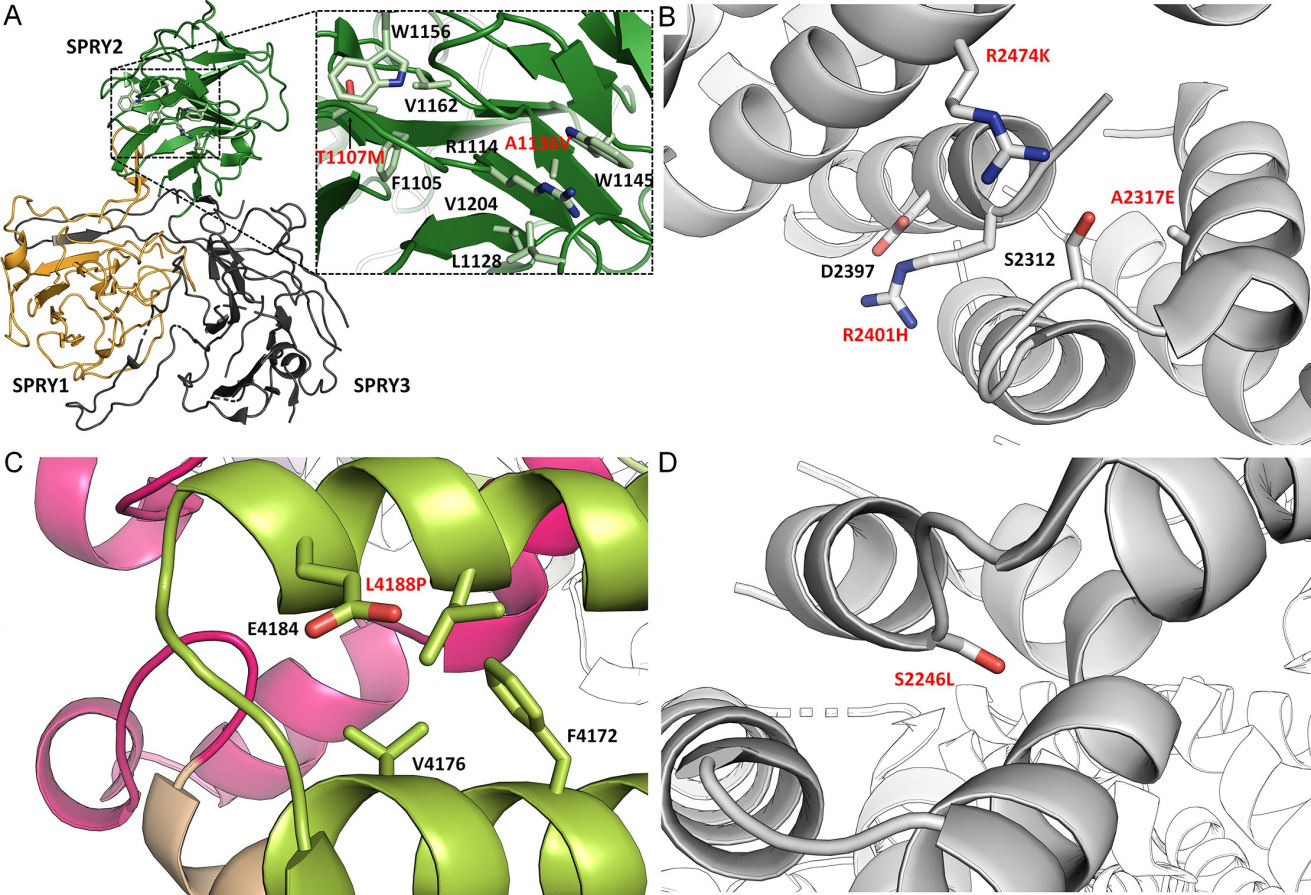
Location of CPVT-associated variants on RyR2 structure (continued). (**A**) *RYR2*-p.T1107M and p.A1136V mutations are located within the SPRY2 domain, where they are buried and surrounded by hydrophobic residues. (**B**) The *RYR2*-p.R2474K, R2401H, and RYR2-A2317E mutants are clustered within the central alpha-solenoid region of RyR2 (Sol2). The R2401 and R2474 potentially interacts with nearby D2397 and S2312 respectively. These mutations are in close proximity to the CSol3 region and the unstructured region of Sol2. This region is intrinsically flexible and generally poorly resolved in the CryoEM map. (**C**) *RYR2*-p.L4188P mutation is located in the TaF domain where it is surrounded by hydrophobic residues. (**D**) *RYR2*-p.S2246L mutation is located in the better resolved region of Sol2, where the side chain is tightly packed against neighbouring alpha helix.

Based on American College of Medical Genetics and Genomics (ACMG) criteria, 13 of 29 variants (45%) were defined as pathogenic/likely pathogenic (P/LP) after a review of the literature and population allele frequencies in the Exome Aggregation Consortium (ExAC) browser [16]. A remaining 16 (65%) were variants of unknown significance (VUS) by ACMG criteria. Table 2 and the supplemental (S2 File) summarize the variants in this population and the clinical and molecular data to support variant pathogenicity using our stepwise approach to classification. We then undertook a detailed analysis of *RYR2* variants using the 3D structure of pig and mouse RyR2 [15, 17] (Figs 1 & 2) based on several rationales. Firstly, predicting the pathogenicity of variants on sequence alone does not consider the chemical environment of the affected residues. Substitutions of amino acid residues involved in protein folding, domain-domain interactions, and interactions with auxiliary ligands are much more likely to affect function than residues simply pointing to solvent. This type of information is not available from sequence-based algorithms like the Polyphen score. Secondly, knowledge of the 3D environment can give possible clues on the disease mechanism. [18] Of 21 *RYR2* variants identified in this study, 12 could be mapped on the open-state structure of RyR2 (Figs 2 and 3), and 11 would likely have a damaging effect on channel function (p.R417L, p.R485Q, p.S3938R, p.K4594R, p.Y4721C, p.S2246L, p.H2464D, p.R2401H, p.L4188P, p.A2317E and p.T1107M). Of note, 2 of these were initially classified as VUS by ACMG criteria (p.L4188P and p.A2317E). In contrast, one *RYR2* pathogenic variant (p.A1136V) and one VUS (p.R2028H) by ACMG standards appeared benign based on structural mapping and sequence conservation. The model for each of these variants appear in Figs 2 & 3. The supplemental (S2 File) provides all the original data for the classification of each variant.

## Discussion

In this study, multi-variant CPVT occurred in 8% of the PACES CPVT Registry. Nearly three-quarters of these children were SCA survivors and many had variants that were likely pathogenic. While this cohort of multi-variant CPVT patients were severely affected, there are insufficient data to conclude whether this genetic phenomenon has prognostic implications. Even in the absence of prognostic utility, multi-variant CPVT is relevant to decision-making around cascade family screening. In total, there were 3 possible situations observed: (1) double variants in cis, (2) compound heterozygous variants in trans, and (3) digenic heterozygous variants (different genes). As will be discussed, each scenario creates a unique set of diagnostics, cascade screening and prognostic implications.

The cumulative gene dosage phenomenon is similarly rare for hypertrophic cardiomyopathy, [9, 10] arrhythmogenic right ventricular cardiomyopathy, [11] and LQTS [8, 12], compared to CPVT in the PACES Registry. Multi-variant CPVT children were usually severely affected (73% with SCA). In comparison to the rest of the Registry population (38% with SCA), the severity of multi-variant CPVT appeared greater, but these data are not sufficient to imply that disease severity is directly influenced by the number of mutations. Statistical comparisons between single vs. multiple variant phenotypes were not undertaken due to the relatively small population described here, and the historical uncertainty around the genetic testing protocols/techniques which pre-dated enrollment in the registry. For example, early on, commercial genetic testing companies were only testing select *RYR2* exons and older reports in the Registry were sometimes incomplete. Additionally, the second variant was sometimes a VUS, and/or potentially benign based on our mapping and/or found in cis phase. In such settings, CPVT may be driven by a single variant or the second variant could be a risk modifier. An example is *RYR2-*p.G1886S, which occurred in 2 of our patients, and was also seen in the general population. [16] A recent study has shown that this variant is a significant risk factor for ventricular arrhythmias in heart failure patients [21], suggesting that it could be a candidate risk modifier in CPVT. A parallel effect exists in the LQTS genes where common variants underlie a susceptibility to drug-induced QT prolongation [22, 23] but do not cause overt LQTS. For example, *KCNE1-*p.D85N is too common in the population to independently cause LQTS, but significantly increases risk if a second LQTS mutation occurs. [24, 25] In our cohort, subject #14 carried a *CACNA1C* VUS and a pathogenic *RYR2* mutation (plus several other *RYR2* VUS), and had a classic CPVT phenotype (catecholamine triggered bidirectional VT) with some QT prolongation. We hypothesize that a possible LQTS variant, plus multiple variants linked to CPVT, may have collectively contributed to his severe overlap phenotype (ie. digenic heterozygosity). Quite remarkably, another boy had two forms of CPVT (type-1 due to *RYR2-*p.T1107M and type-2 due to *CASQ2-*c.IVS5+1G>C). While CPVT type-2 alone can be especially dangerous, the *RYR2-*p.T1107M variant also likely has a damaging role based on the present data, and previous studies showing both a clinical and *in vitro* phenotype [19, 26]. The growing international CPVT registry and prospective data are needed to clarify the risk and incidence of multi-variant CPVT.

The presence ≥2 variants creates other logistical problems. We could not differentiate between cis and trans variants in some cases owing to the inconsistent screening of the parents. Incomplete parental screening may be due to the clinicians’ uncertainty regarding the disease-causing variant in the family, thus demonstrating another challenge in this circumstance. Variants in cis phase similarly confounds screening in hypertrophic cardiomyopathy. [27] In cis phase, the CPVT phenotype would not necessarily be worse than any given single variant. However, the presence of ≥2 cis variants is relevant, as it demonstrates the complexities around family screening in the setting of CPVT. *RYR2* variants in trans phase have a theoretical mechanism for increased severity. RyR2 is a large, homotetrameric protein made up of 4 subunits. Two variants in trans phase would mean that all 4 subunits making up the channel would be mutated. In contrast, in the typical case of autosomal dominant CPVT, half of the four subunits would be wildtype. Theoretically, this could account for a more severe phenotype in compound multi-variant CPVT. Based on the present study, we propose that targeted sequencing for both variants be performed in the clinical setting, and that relatives, especially parents, need to be evaluated by an expert to clarify the role of each variant.

The unclear pathogenicity of *RYR2* variants is a growing concern in CPVT. [28] We used a standardized bioinformatics approach to variant interpretation to avoid overcalling pathogenicity. After applying the ACMG criteria, we mapped variants on the open-state structure of RyR2 to see if any other insights could be obtained. This technique relies on the 3D structure derived from high resolution studies of the ryanodine receptor. A good example of this is the N-terminal region of RyR2, which consists of three domains: domain A (residues 1–217) and domain B (residues 218–409), and domain C (residues 410–543) (Fig 1). [29–31] A chloride ion is coordinated by residues of all three domains and disruption of this binding site *via* disease-causing variants results in domain reorientations. [17, 29, 32] These observations suggest that CPVT variants may destabilize domain interfaces or disrupt the folding of individual domains, which would impair domain-domain interactions and cause adverse effects on channel gating. [17, 20, 29, 31–34] Structural analyses supported downgrading p.A1136V and p.R2028H to likely benign. *RYR2-*p.A1136 is located in a relatively non-conserved region within the SPRY2 domain, where its equivalent residue in both RyR1 and RyR3 is a valine, thus substitution to valine is unlikely to alter the function of RyR2 significantly. Further, structural mapping showed that valine substitution can be easily accommodated without the formation of steric clashes. The p.R2028H mutation is located in a flexible region of RyR2, where the side chain is pointing towards the solvent, and thus the mutation is unlikely to have a major impact on channel function. These are not functional assays, so the conclusions are predictive in nature.

This study is limited by its retrospective design. Genetic testing spanned nearly 15 years, and not all commercial testing companies provided technical details as would be required in the contemporary era. Results for family members were sometimes not available (often if followed by a non-participating center). Early commercial sequencing methods could not differentiate between two variants in the cis vs. trans position of *RYR2* (unpublished communication). Limitations also exist in the structural analysis of variants, whereby some portions of RyR2 structure are poorly defined in the CryoEM structure. The best-defined regions are domains whose structures have been determined *via* X-ray crystallography (N-terminal domains, SPRY1/2, Rep12, and Rep34 domains), followed by the C-terminal and transmembrane regions, for which the resolution of CryoEM studies is the highest [18]. As such, direct analysis of variants in the N-terminal and C-terminal hotspots is the most reliable. For most other sections, direct analysis of hydrogen bonds and ionic interactions of the variants is not yet possible, however their general location in the 3D structure can be determined at the current resolution for RyR2. A detailed supplemental disclosing all the supporting data is provided to facilitate re-classification by future researchers as the field advances.

## Conclusions

More than one variant may underlie a minority of CPVT cases. This poses challenges with respect to diagnosis and family counselling. While multi-variant CPVT patients were usually severely affected, further research is needed to determine the significance and generalizability of this observation. We demonstrate that a rigorous approach to variant re-classification using the ACMG criteria and 3D mapping is important in reaching an accurate diagnosis, especially in the multiple variant population.

## Supporting information

S1 File Detailed material and methodology. (DOCX)

S2 File Detailed classification scheme for all variants in the population. (DOCX)

S3 File Pedigrees of select multi-variant families. (DOCX)
