# Supplementary material for: Catecholaminergic polymorphic ventricular tachycardia patients with multiple genetic variants in the PACES CPVT Registry
Source: PLoS One. 2018 Nov 7;13(11):e0205925. doi: 10.1371/journal.pone.0205925 (PMC6221297; doi:10.1371/journal.pone.0205925)
Supplement: S1 File — (DOCX) [file pone.0205925.s001.docx]

**Supplemental Information**

**S1 File: Detailed Material and Methods**

**Clinical Analysis & Definitions:**

Genetic testing was performed for routine patient care and was therefore not standardized. Patients who had ≥2 variants in CPVT-associated gene(s) reported to be pathogenic, likely pathogenic (P/LP) and/or variant(s) of uncertain pathogenicity (VUS) were included. Data from medical records were inputted by individual sites in a secure online database (REDCap – Research Electronic Data Capture), (1) which was hosted and overseen by the coordinating center (British Columbia Children’s Hospital). A proband was defined as the index case of confirmed catecholaminergic polymorphic ventricular tachycardia (CPVT) in a family, and treatment failure was defined as sudden cardiac arrest, syncope and/or appropriate implantable cardioverter defibrillator shock while on pharmacologic therapy. Duration of follow-up was calculated as the time from initial diagnosis/sentinel CPVT symptom to most recent known cardiology assessment.

**Genetic Analysis and Pathogenicity Classification:**

A systematic, stepwise approach to variant classification was undertaken. The commercial lab interpretation of pathogenicity was initially recorded in the database. While the terminology used by laboratories has become more standardized in recent years, for earlier reports, commercial companies used variable terminology to define variant pathogenicity. We often needed to update their terminology using the following modern classification scheme: pathogenic, likely pathogenic (grouped together as P/LP) or VUS. The most common historical term used by laboratories was the variant of “possible” pathogenicity. We re-classified this as a P/LP, given that the commercial testing company using this term also had a separate category for VUS. These commercial interpretations were then re-classified using the American College of Medical Genetics and Genomics (ACMG)(2) criteria, followed by 3D mapping using an RyR2 model. To apply the ACMG criteria, we first accessed the Exome Aggregation Consortium (ExAC) browser determine the population frequency of each variant. ExAC is an online repository of genetic information from 60,706 unrelated individuals sequenced as part of various disease-specific and population studies.(3) Next, we performed a detailed literature review using PubMED, Google Scholar, and the bibliographies of major papers in the field to identify any clinical and functional data on candidate mutations. After this process, variants were then re-classified according to contemporary criteria for pathogenicity defined by the ACMG(2), which require the latter two steps to undertake a complete assessment of a variant. We then used the 3D model of pig (*Sus scrofa*) RyR2 in the open-state (PDB ID: 5GOA) to predict structural impact of variants whenever possible, which can inform the underlying mechanism behind CPVT.(4) Analysis of the 3D structural environment of all variants was performed using Pymol (v2.1Schrödinger, LLC.). The supplemental tables below summarize the criteria applied for determination of pathogenicity, including the results of the literature review, ExAC allele frequencies and 3D structural mapping.

**S1 File Supplemental References:**

1. Harris PA, Taylor R, Thielke R, Payne J, Gonzalez N, Conde JG. Research Electronic Data Capture (REDCap) - A metadata-driven methodology and workflow process for providing translational research informatics support. Journal of biomedical informatics. 2009;42(2):377-81.

2. Richards S, Aziz N, Bale S, Bick D, Das S, Gastier-Foster J, et al. Standards and guidelines for the interpretation of sequence variants: a joint consensus recommendation of the American College of Medical Genetics and Genomics and the Association for Molecular Pathology. Genetics in medicine : official journal of the American College of Medical Genetics. 2015;17(5):405-24.

3. Lek M, Karczewski KJ, Minikel EV, Samocha KE, Banks E, Fennell T, et al. Analysis of protein-coding genetic variation in 60,706 humans. Nature. 2016;536(7616):285-91.

4. Roston TM, Yuchi Z, Kannankeril PJ, Hathaway J, Vinocur JM, Etheridge SP, et al. The clinical and genetic spectrum of catecholaminergic polymorphic ventricular tachycardia: findings from an international multicentre registry. Europace : European pacing, arrhythmias, and cardiac electrophysiology : journal of the working groups on cardiac pacing, arrhythmias, and cardiac cellular electrophysiology of the European Society of Cardiology. 2017.
