# Supplementary material for: Catecholaminergic polymorphic ventricular tachycardia patients with multiple genetic variants in the PACES CPVT Registry
Source: PLoS One. 2018 Nov 7;13(11):e0205925. doi: 10.1371/journal.pone.0205925 (PMC6221297; doi:10.1371/journal.pone.0205925)
Supplement: S2 File — (DOCX) [file pone.0205925.s002.docx]

**Supplemental Information:**

**S2 File: Dataset**

**Table:** Detailed classification scheme for all variants in the population

| **Variant** | **Consequence** | **Reported pathogenicity*** | **Published evidence** | **First author of previous reports** | **ExAC Allele Frequency^1^** | **ACMG Criteria^2^** | **ACMG Conclusion** | **Structural modeling in present study** | **Updated pathogenicity based on modeling vs. ACMG criteria** |
| --- | --- | --- | --- | --- | --- | --- | --- | --- | --- |
| *RYR2*-p.R417L | Missense | P/LP | No reports | N.A. | Absent | Moderate (PM1, PM2, PM6); Supporting (PP2, PP4) | Likely pathogenic | R417 is located inside an alpha helix in domain C at domains A-C and B-C interfaces, near the anion-binding site. The inter-domain area is dominated by hydrophilic and charged residues. The R417L mutation would introduce a shorter, hydrophobic side chain in place of a bulky, positively charged side chain, which may alter the anion binding and cause domain-domain rearrangements. | Unchanged |
| *RYR2*-p.F3496L | Missense | VUS | No reports | N.A. | Absent | Moderate (PM2, PM6); Supporting (PP1, PP2, PP4) | Likely pathogenic | F3496 is located in an intrinsically disordered alpha-solenoidal region of RyR2 (Sol2), and can thus not be visualized. It is currently unknown whether any auxiliary protein binds to this region. | Unchanged |
| *RYR2-*p.S3938R | Missense | P/LP | 2 cases; no co-segregation or in vitro testing reported | Medeiros-Domingo et al.^3^  Tester et al.^4^ | Absent | Moderate (PM1, PM2, PM6); Supporting (PP2, PP4) | Likely pathogenic | S3938 is located in the CSol3 region of RyR2. S3938 is near the pore, within the cytosolic side of the channel. Mutation to bulkier, positively charged side chain may alter hydrogen bonding pattern at this site and/or disrupt surrounding alpha helices structure. Due to the large open space surrounding this residue, the site may also influence binding to an unknown auxiliary protein. | Unchanged |
| *RYR2-*p.R485Q | Missense | VUS | Structural modeling showing loss of π-cation interaction | Bottillo et al.^5^ | 0.00008645 | Moderate (PM1, PM6); Supporting (PP2, PP4) | Likely pathogenic | R485 is located inside an alpha helix of domain C, buried within the helical bundle. The R485 side chain forms a salt bridge with the E411, located in another helix facing domains A and B. The R485Q mutation would break this interaction, destabilizing domain C, and affect the anion binding site. | Unchanged |
| *RYR2-*p.R2474K | Missense | P/LP | 1 case; no co-segregation or in vitro testing reported | Kozlovski et al.^6^ | Absent | Moderate (PM1, PM2, PM6); Supporting (PP2) | Likely pathogenic | R2474 is located in the Sol2 region of RyR2. Region is poorly resolved in CryoEM structures. The variant is subtle and structural predicted suggests a minimal impact. It is currently unknown whether any auxiliary protein binds to this region. | Unchanged |
| *RYR2-*p.A1136V | Missense | VUS | 1 case reported^3^, present in healthy controls^7^; no co-segregation or in vitro testing reported | Medeiros-Domingo et al.^3^  Kaartinen et al.^7^ | 0.007063 | Strong (PS2); Supporting (PP2, PP4) | Likely pathogenic | A1136 is located within the SPRY2 domain. It is buried inside a hydrophobic core, and a substitution to a larger side chain may perturb the folding and form clashes with nearby residues such as R1114 and L1128. The equivalent residue in both RyR1 and RyR3 is a valine, therefore the mutation is unlikely to have significant negative impact on the overall structure of RyR. | Downgraded |
| *RYR2-*p.I2075T | Missense | P/LP | Same family previously published; no co-segregation or in vitro testing reported | Paech et al.^8^ | 0.000009395 | Supporting (PP1, PP2, PP4) | VUS | I2075 is located within an alpha-solenoid region, where it is buried between two helices. Substitution by Thr is likely to affect the helical packing. Importantly, it is very close to an interface with another alpha solenoid region, and the variant may thus impact this interdomain interaction. | Unchanged |
| *RYR2-*p.K4594R | Missense | VUS | Same family previously published; no co-segregation or in vitro testing reported | Paech et al.^8^ | Absent | Moderate (PM1, PM2); Supporting (PP1; PP2; PP4) | Likely pathogenic | K4594 is located at the cytosolic edge of the pseudo voltage-sensing domain (pVSD), with potential interactions with the thumb and forefingers (TaF) domain. These domains are implicated in the binding of activating ligands and channel opening. Although the K4594R substitution is conservative, the guanidinium group of Arg allows for a larger number of electrostatic interactions. Further, the higher pKa of the guanidinium group may facilitate a stronger interaction with nearby E4200, which could impact on the ATP/Caffeine binding sites located nearby (Fig. 2H). Thus any small perturbation in this area is likely to alter channel gating. | Unchanged |
| *RYR2-*p.R2028H | Missense | P/LP | No reports | N.A. | Absent | Moderate (PM2); Supporting (PP2, PP4) | VUS | R2028 is found in Sol2 region of RyR2, where the side chain is pointing toward the solvent. The variant is unlikely to have a major impact on the function, but may influence binding to an unknown auxiliary protein. | Downgraded |
| *RYR2-*p.Y4721C | Missense | P/LP | No reports | N.A. | Absent | Moderate (PM1, PM2); Supporting (PP2, PP4) | Likely pathogenic | This residue is located within the transmembrane region of pVSD. This region plays an important role in allosteric gating of the channel and the Tyr is surrounded by other hydrophobic residues. Mutation to cysteine is likely to perturb channel gating and domain packing. | Unchanged |
| *RYR2-*p.L4188P | Missense | VUS | Study subject also reported previously^9^; Polyphen-2 score consistent with probable damaging effect but present in population^10^ | Jabbari et al.^10^  LaPage et al.^9^ | Absent | Strong (BS2);  Moderate (PM1, PM2, PM6); Supporting (PP2; PP4) | VUS | L4188 is located within the TaF domain that clamps the C-terminal extension of the RyR. This interaction is critical for channel gating. The substitution by Pro, which promotes helix breaking, and may perturb channel gating. | Upgraded |
| *RYR2-*p.H2464D | Missense | P/LP | 1 case; human derived pluripotent stem cells showing gain of function^11^ | Hernandez et al.^11^ | Absent | Strong (PS3); Moderate (PM1, PM2, PM6); Supporting (PP2, PP4) | Pathogenic | H2464 is located within a poorly resolved Sol2 region of RyR2 structure. The variant may impact binding of an unknown auxiliary protein to this region. | Unchanged |
| *RYR2-*p.S2246L | Missense | P/LP | Reported in multiple human CPVT patients^3, 12^; augmented calcium release to beta-adrenergic agents in HL-1 derived cardiomyocytes;^13^ Knock-in mouse model showing increase calcium spark frequency and lethal arrhythmia^14^ | George et al.^13^  Suetomi et al.^14^  Medeiros-Domingo et al.^3^  Postma et al.^12^ | Absent | Strong (PS3); Moderate (PM1, PM2, PM6); Supporting (PP2; PP4) | Pathogenic | S2246 is located within the Sol2 region, where the side chain is tightly packed next to an alpha helix. Mutation to a longer side chain likely results in steric clashes, and will impact helix packing in this region.. | Unchanged |
| *RYR2-*p.G1885E | Missense | VUS | 3 cases of compound heterozygous (trans) ARVC with *RYR2-*G1886S^15^ and two ARVC families with double mutations (cis) with *RYR2-Q*2958R^16^; HEK293 mutants demonstrate gain of function^17^ | Milting et al.^15^  Tiso et al.^16^  Koop et al.^17^ | 0.01540 | Strong (PS3, BS1; BS2); Supporting (PP2, PP4) | VUS | G1885 is located in a flexible unstructured loop. Though the substitution is unlikely to have an impact on channel gating, because this region is highly conserved among in RyRs, the region may play an important allosteric role or is a part of a binding site for an auxiliary protein. | Unchanged |
| *RYR2-*p.G1886S | Missense | VUS | 3 cases of compound heterozygous (trans) ARVC with *RYR2-*G1885E^15^ and associated with increased susceptibility in population of ICD patients with heart failure^18^ | Milting et al.^15^  Francia et al.^18^ | 0.04385 | Strong (BS1, BS1); Supporting (PP2, PP4) | VUS | G1886 is located in a flexible unstructured loop as part of Sol2 region. Though the substitution alone is unlikely to have an impact on channel gating, it may have indirect effects such as creation of a new phosphorylation site. | Unchanged |
| *RYR2-*p.T1107M | Missense | VUS | 1 family with hypertrophic cardiomyopathy^19^; HEK293 mutants demonstrate loss of function^20^ and structural modeling shows steric clash with neighboring hydrophobic residues^21^ | Noboru et al.^19^  Tang et al.^20^  Lau et al.^21^ | 0.0003230 | Strong (PS2, PS3); Supporting (PP2, PP3, PP4) | Pathogenic | T1107 is located within the SPRY2 domain, where it is buried and surrounded by hydrophobic residues. The mutation would form steric clashes with W1156 and cause destabilization of the domain. Functional experiments have shown it affects Ca^2+^ release properties. | Unchanged |
| *RYR2-*p.G4772S | Missense | P/LP | No reports | N.A. | Absent | Moderate (PM2); Supporting (PP1, PP2, PP4) | VUS | G4772 is located in the pore forming domain (PFD), as part of the outer helix. Substitution to less flexible Ser may affect helical packing within the membrane and cause subtle domain rearrangements. | Unchanged |
| *RYR2-*p.R2401H | Missense | P/LP | Several isolated cases^22-24^ | Aizawa et al.^24^  Liu et al.^22^  Creighton et al.^23^ | Absent | Moderate (PM1, PM2); Supporting (PP2, PP4) | Likely pathogenic | R2401 is located within the Sol2 region. It forms a salt bridge with a neighboring D2397 residue. Substitution to His may have a minor impact on helix stability. | Unchanged |
| *RYR2-*c.3599-9delT | Splice site | VUS | No reports | N.A. | Absent | Moderate (PM2); Supporting (PP4, BP3) | VUS | Not performed | N.A. |
| *RYR2-*c.14091-11dupT | Splice site | VUS | No reports | N.A. | Absent | Moderate (PM2); Supporting (PP4, BP3) | VUS | Not performed | N.A. |
| *RYR2-*p.A2317E | Missense | P/LP | 1 case^25^; Polyphen score consistent with probable damaging effect but present in general population^10^ | Van der Werf et al.^25^  Jabbari et al.^10^ | Absent | Strong (BS3); Moderate (PM1); Supporting (PP2, PP4) | VUS | A2317 is in an alpha solenoid region, where it is involved in packing with neighbouring residues. Mutation to the larger Glu residue likely forms steric clashes with nearby residues, and this is likely to affect packing and stability of the region. | Upgraded |
| *CASQ2-*IVS5+1G>C | Splice site | P/LP | No reports | N.A. | Absent | Very Strong (PVS1); Moderate (PM2); Supporting (PP4, PP5) | Pathogenic | Not performed | N.A. |
| *SCN5A-*p.Q692K | Missense | P/LP | 1 sudden infant death case^26^ but reclassified as polymorphism based on population data^27^ | Millat et al.^26^  Kapplinger et al.^27^ | 0.0002822 | Strong (BS2); Supporting (PP4) | VUS | Not performed | N.A. |
| *DSG-*p.V288I | Missense | VUS | No reports | N.A. | Absent | Moderate (PM2); Supporting (PP4) | VUS | Not performed | N.A. |
| *CACNA1C-*p.T1870M | Missense | VUS | No reports | N.A. | Absent | Moderate (PM2); Supporting (PP2, PP4) | VUS | Not performed | N.A. |
| *CACNA1C-*c.5680+11C>T | Intronic | VUS | No reports | N.A. | Absent | Moderate (PM2); Supporting (PP4, BP3) | VUS | Not performed | N.A. |
| *TMEM43-*c.512+19G>T | Intronic | VUS | 1 case of ARVC^28^ | Haywood et al.^28^ | Absent | Moderate (PM2); Supporting (PP4, BP3) | VUS | Not performed | N.A. |
| *PKP2-*c.2300-4G>C | Splice site | VUS | No reports | N.A. | 0.00008079 | Supporting (PP4, BP3) | VUS | Not performed | N.A. |
| *DSP-*p.R1458G | Missense | P/LP | 1 ARVC^29^ and 1 dilated cardiomyopathy^30^ patient, both with additional VUS | Cox et al.^29^  Pugh et al.^30^ | 0.001737 | Supporting (PP4) | VUS | Not performed | N.A. |

* P/LP=pathogenic/likely pathogenic; ACMG=American College Medical Genetics; N.A.=not applicable; VUS=variant of uncertain significance

**S2 File Supplemental References:**

1. Lek M, Karczewski KJ, Minikel EV, Samocha KE, Banks E, Fennell T, O’Donnell-Luria AH, Ware JS, Hill AJ, Cummings BB, Tukiainen T, Birnbaum DP, Kosmicki JA, Duncan LE, Estrada K, Zhao F, Zou J, Pierce-Hoffman E, Berghout J, Cooper DN, Deflaux N, DePristo M, Do R, Flannick J, Fromer M, Gauthier L, Goldstein J, Gupta N, Howrigan D, Kiezun A, Kurki MI, Moonshine AL, Natarajan P, Orozco L, Peloso GM, Poplin R, Rivas MA, Ruano-Rubio V, Rose SA, Ruderfer DM, Shakir K, Stenson PD, Stevens C, Thomas BP, Tiao G, Tusie-Luna MT, Weisburd B, Won H-H, Yu D, Altshuler DM, Ardissino D, Boehnke M, Danesh J, Donnelly S, Elosua R, Florez JC, Gabriel SB, Getz G, Glatt SJ, Hultman CM, Kathiresan S, Laakso M, McCarroll S, McCarthy MI, McGovern D, McPherson R, Neale BM, Palotie A, Purcell SM, Saleheen D, Scharf JM, Sklar P, Sullivan PF, Tuomilehto J, Tsuang MT, Watkins HC, Wilson JG, Daly MJ, MacArthur DG and Exome Aggregation C. Analysis of protein-coding genetic variation in 60,706 humans. *Nature*. 2016;536:285-291.

2. Richards S, Aziz N, Bale S, Bick D, Das S, Gastier-Foster J, Grody WW, Hegde M, Lyon E, Spector E, Voelkerding K and Rehm HL. Standards and guidelines for the interpretation of sequence variants: a joint consensus recommendation of the American College of Medical Genetics and Genomics and the Association for Molecular Pathology. *Genetics in Medicine*. 2015;17:405-24.

3. Medeiros-Domingo A, Bhuiyan ZA, Tester DJ, Hofman N, Bikker H, van Tintelen JP, Mannens MM, Wilde AA and Ackerman MJ. The RYR2-encoded ryanodine receptor/calcium release channel in patients diagnosed previously with either catecholaminergic polymorphic ventricular tachycardia or genotype negative, exercise-induced long QT syndrome: a comprehensive open reading frame mutational analysis. *Journal of the American College of Cardiology*. 2009;54:2065-74.

4. Tester DJ, Arya P, Will M, Haglund CM, Farley AL, Makielski JC and Ackerman MJ. Genotypic heterogeneity and phenotypic mimicry among unrelated patients referred for catecholaminergic polymorphic ventricular tachycardia genetic testing. *Heart Rhythm*. 2006;3:800-5.

5. Bottillo I, D’Angelantonio D, Caputo V, Paiardini A, Lipari M, De Bernardo C, Majore S, Castori M, Zachara E, Re F and Grammatico P. Prediction and visualization data for the interpretation of sarcomeric and non-sarcomeric DNA variants found in patients with hypertrophic cardiomyopathy. *Data in Brief*. 2016;7:607-613.

6. Kozlovski J, Ingles J, Connell V, Hunt L, McGaughran J, Turner C, Davis A, Sy R and Semsarian C. Delay to diagnosis amongst patients with catecholaminergic polymorphic ventricular tachycardia. *International Journal of Cardiology.* 2014;176:1402-4.

7. Kaartinen M, Helio T, Lehtonen A, Lahtinen AM, Karkkainen S, Keto P, Kontula K and Toivonen L. Characterization of familial and sporadic arrhythmogenic right ventricular cardiomyopathy in Finland. *Annals of Medicine*. 2007;39:312-8.

8. Paech C, Gebauer RA, Karstedt J, Marschall C, Bollmann A and Husser D. Ryanodine receptor mutations presenting as idiopathic ventricular fibrillation: a report on two novel familial compound mutations, c.6224T>C and c.13781A>G, with the clinical presentation of idiopathic ventricular fibrillation. *Pediatric Cardiology*. 2014;35:1437-41.

9. LaPage MJ, Russell MW, Bradley DJ and Dick M, 2nd. Novel ryanodine receptor 2 mutation associated with a severe phenotype of catecholaminergic polymorphic ventricular tachycardia. *J Pediatr*. 2012;161:362-4.

10. Jabbari J, Jabbari R, Nielsen MW, Holst AG, Nielsen JB, Haunso S, Tfelt-Hansen J, Svendsen JH and Olesen MS. New exome data question the pathogenicity of genetic variants previously associated with catecholaminergic polymorphic ventricular tachycardia. *Circulation Cardiovascular Genetics*. 2013;6:481-9.

11. Hernández JJ, Herron T, Jalife J, Maginot K, Zhang J, Kamp T and Valdivia HH. Abstract 17750: A CPVT Mutation Confers Gain of Function to the Cardiac Ryanodine Receptor Channel. Characterization Using Cardiomyocytes Derived From Patient-Specific Ips Cells. *Circulation*. 2016;128:A17750.

12. Postma AV, Denjoy I, Kamblock J, Alders M, Lupoglazoff JM, Vaksmann G, Dubosq-Bidot L, Sebillon P, Mannens MM, Guicheney P and Wilde AA. Catecholaminergic polymorphic ventricular tachycardia: RYR2 mutations, bradycardia, and follow up of the patients. *J Med Genet*. 2005;42:863-70.

13. George CH, Higgs GV and Lai FA. Ryanodine receptor mutations associated with stress-induced ventricular tachycardia mediate increased calcium release in stimulated cardiomyocytes. *Circulation Research*. 2003;93:531-40.

14. Suetomi T, Yano M, Uchinoumi H, Fukuda M, Hino A, Ono M, Xu X, Tateishi H, Okuda S, Doi M, Kobayashi S, Ikeda Y, Yamamoto T, Ikemoto N and Matsuzaki M. Mutation-linked defective interdomain interactions within ryanodine receptor cause aberrant Ca(2)(+)release leading to catecholaminergic polymorphic ventricular tachycardia. *Circulation*. 2011;124:682-94.

15. Milting H, Lukas N, Klauke B, Körfer R, Perrot A, Osterziel K-J, Vogt J, Peters S, Thieleczek R and Varsányi M. Composite polymorphisms in the ryanodine receptor 2 gene associated with arrhythmogenic right ventricular cardiomyopathy. *Cardiovascular Research*. 2006;71:496-505.

16. Tiso N, Stephan DA, Nava A, Bagattin A, Devaney JM, Stanchi F, Larderet G, Brahmbhatt B, Brown K, Bauce B, Muriago M, Basso C, Thiene G, Danieli GA and Rampazzo A. Identification of mutations in the cardiac ryanodine receptor gene in families affected with arrhythmogenic right ventricular cardiomyopathy type 2 (ARVD2). *Human Molecular Genetics*. 2001;10:189-94.

17. Koop A, Goldmann P, Chen SR, Thieleczek R and Varsanyi M. ARVC-related mutations in divergent region 3 alter functional properties of the cardiac ryanodine receptor. *Biophysical Journal*. 2008;94:4668-77.

18. Francia P, Adduci C, Semprini L, Stanzione R, Serdoz A, Caprinozzi M, Santini D, Cotugno M, Palano F, Musumeci MB, Rubattu S and Volpe M. RyR2 Common Gene Variant G1886S and the Risk of Ventricular Arrhythmias in ICD Patients with Heart Failure. *Journal of Cardiovascular Electrophysiology*. 2015;26:656-61.

19. Fujino N, Ino H, Hayashi K, Uchiyama K, Nagata M, Konno T, Katoh H, Sakamoto Y, Tsubokawa T, Ohsato K, Mizuno S and Yamagishi M. Abstract 915: A Novel Missense Mutation in Cardiac Ryanodine Receptor Gene as a Possible Cause of Hypertrophic Cardiomyopathy: Evidence From Familial Analysis. *Circulation*. 2015;114:II_165.

20. Tang Y, Tian X, Wang R, Fill M and Chen SR. Abnormal termination of Ca2+ release is a common defect of RyR2 mutations associated with cardiomyopathies. *Circulation Research*. 2012;110:968-77.

21. Lau K and Van Petegem F. Crystal structures of wild type and disease mutant forms of the ryanodine receptor SPRY2 domain. *Nature Communications*. 2014;5:5397.

22. Liu X LJ, Shen Y, Wan R, Xiong QM, Zhou QQ, Xie JY, Jin JJ, Yan X, Yu JH, Hong K. Recurrent Syncope Related to Catecholaminergic Polymorphic Ventricular Tachycardia Due to De Novo RyR2-R2401H Mutation. *Zhonghua Xin Xue Guan Bing Za Zhi*. 2017;45:39-43.

23. Creighton W, Virmani R, Kutys R and Burke A. Identification of Novel Missense Mutations of Cardiac Ryanodine Receptor Gene in Exercise-Induced Sudden Death at Autopsy. *The Journal of Molecular Diagnostics*. 2006;8:62-67.

24. Aizawa Y, Ueda K, Komura S, Washizuka T, Chinushi M, Inagaki N, Matsumoto Y, Hayashi T, Takahashi M, Nakano N, Yasunami M, Kimura A, Hiraoka M and Aizawa Y. A novel mutation in FKBP12.6 binding region of the human cardiac ryanodine receptor gene (R2401H) in a Japanese patient with catecholaminergic polymorphic ventricular tachycardia. *International Journal of Cardiology*. 2005;99:343-5.

25. van der Werf C, Nederend I, Hofman N, van Geloven N, Ebink C, Frohn-Mulder IM, Alings AM, Bosker HA, Bracke FA, van den Heuvel F, Waalewijn RA, Bikker H, van Tintelen JP, Bhuiyan ZA, van den Berg MP and Wilde AA. Familial evaluation in catecholaminergic polymorphic ventricular tachycardia: disease penetrance and expression in cardiac ryanodine receptor mutation-carrying relatives. *Circulation Arrhythmia and Electrophysiology*. 2012;5:748-56.

26. Millat G, Kugener B, Chevalier P, Chahine M, Huang H, Malicier D, Rodriguez-Lafrasse C and Rousson R. Contribution of long-QT syndrome genetic variants in sudden infant death syndrome. *Pediatric Cardiology*. 2009;30:502-9.

27. Kapplinger JD, Tester DJ, Alders M, Benito B, Berthet M, Brugada J, Brugada P, Fressart V, Guerchicoff A, Harris-Kerr C, Kamakura S, Kyndt F, Koopmann TT, Miyamoto Y, Pfeiffer R, Pollevick GD, Probst V, Zumhagen S, Vatta M, Towbin JA, Shimizu W, Schulze-Bahr E, Antzelevitch C, Salisbury BA, Guicheney P, Wilde AAM, Brugada R, Schott J-J and Ackerman MJ. An international compendium of mutations in the SCN5A-encoded cardiac sodium channel in patients referred for Brugada syndrome genetic testing. *Heart Rhythm*. 2010;7:33-46.

28. Haywood AF, Merner ND, Hodgkinson KA, Houston J, Syrris P, Booth V, Connors S, Pantazis A, Quarta G, Elliott P, McKenna W and Young TL. Recurrent missense mutations in TMEM43 (ARVD5) due to founder effects cause arrhythmogenic cardiomyopathies in the UK and Canada. *European Heart Journal*. 2013;34:1002-11.

29. Cox MG, van der Zwaag PA, van der Werf C, van der Smagt JJ, Noorman M, Bhuiyan ZA, Wiesfeld AC, Volders PG, van Langen IM, Atsma DE, Dooijes D, van den Wijngaard A, Houweling AC, Jongbloed JD, Jordaens L, Cramer MJ, Doevendans PA, de Bakker JM, Wilde AA, van Tintelen JP and Hauer RN. Arrhythmogenic right ventricular dysplasia/cardiomyopathy: pathogenic desmosome mutations in index-patients predict outcome of family screening: Dutch arrhythmogenic right ventricular dysplasia/cardiomyopathy genotype-phenotype follow-up study. *Circulation*. 2011;123:2690-700.

30. Pugh TJ, Kelly MA, Gowrisankar S, Hynes E, Seidman MA, Baxter SM, Bowser M, Harrison B, Aaron D, Mahanta LM, Lakdawala NK, McDermott G, White ET, Rehm HL, Lebo M and Funke BH. The landscape of genetic variation in dilated cardiomyopathy as surveyed by clinical DNA sequencing. *Genetics in Medicine*. 2014;16:601-8.
