# Supplementary material for: Catecholaminergic polymorphic ventricular tachycardia patients with multiple genetic variants in the PACES CPVT Registry
Source: PLoS One. 2018 Nov 7;13(11):e0205925. doi: 10.1371/journal.pone.0205925 (PMC6221297; doi:10.1371/journal.pone.0205925)
Supplement: S3 File — (DOCX) [file pone.0205925.s003.docx]

**Supplemental Information:**

**S3 File: Pedigrees**


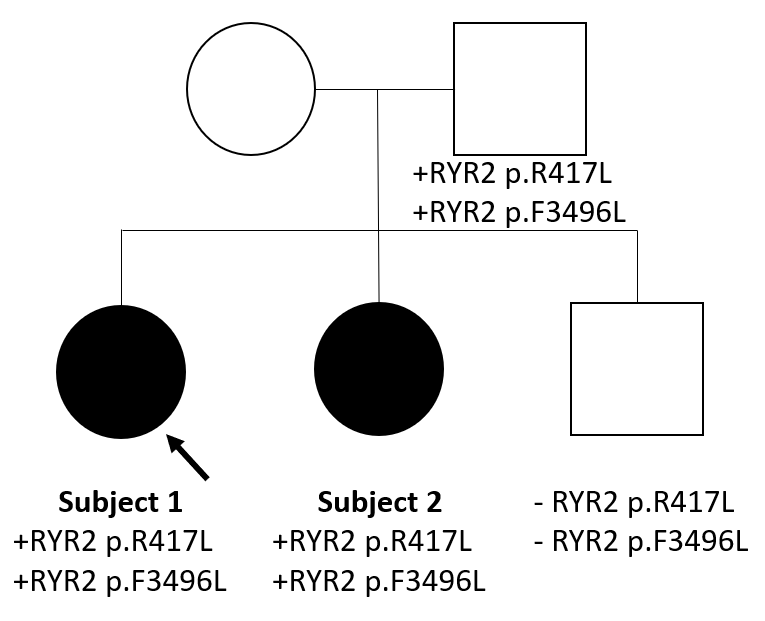


Pedigree 1: Family of Subjects 1 and 2


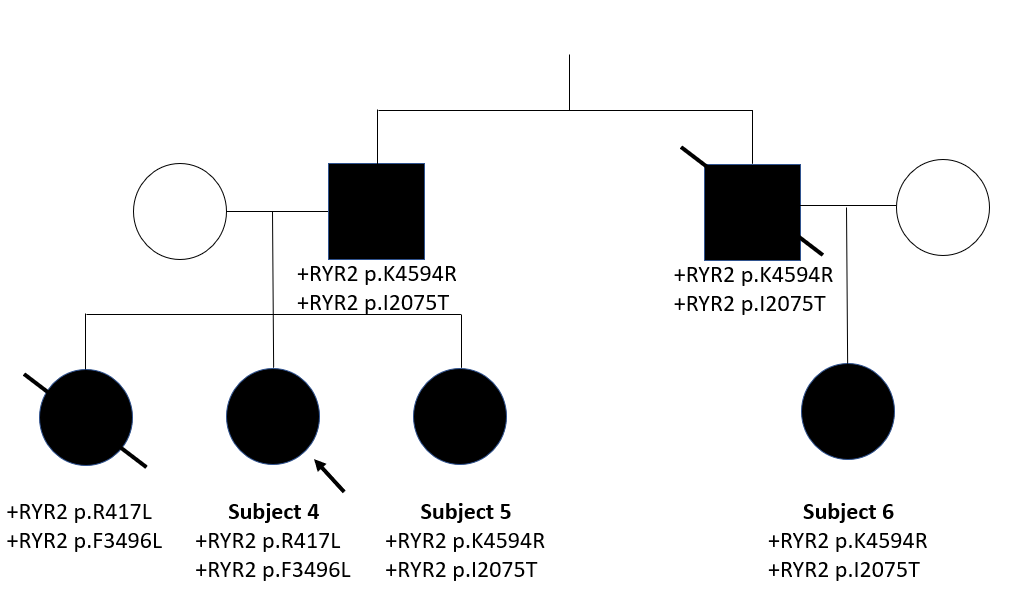


Pedigree 2: Family of Subjects 4, 5 & 6


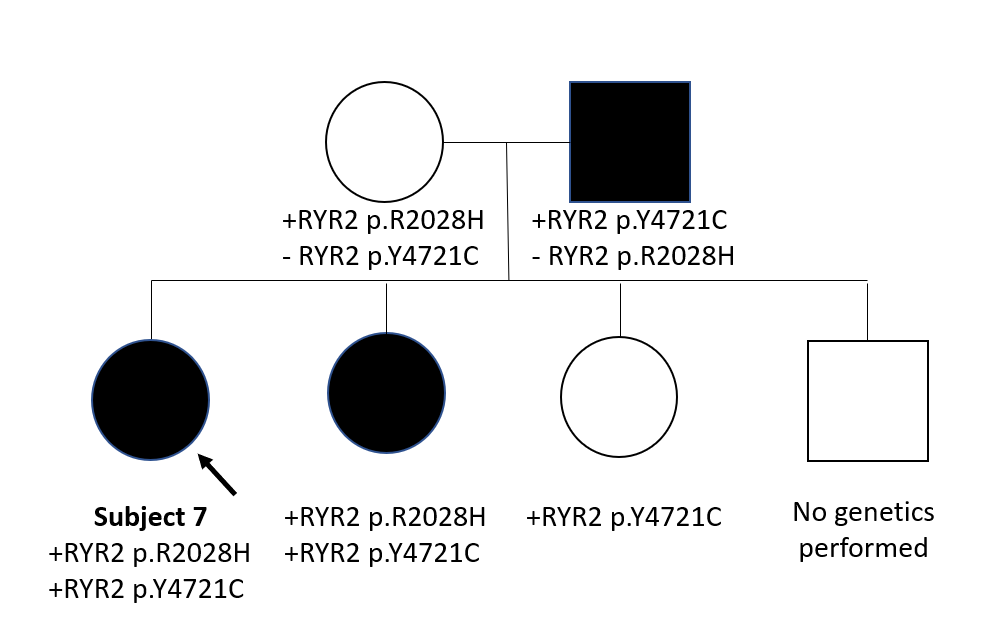


Pedigree 3: Family of Subject 7


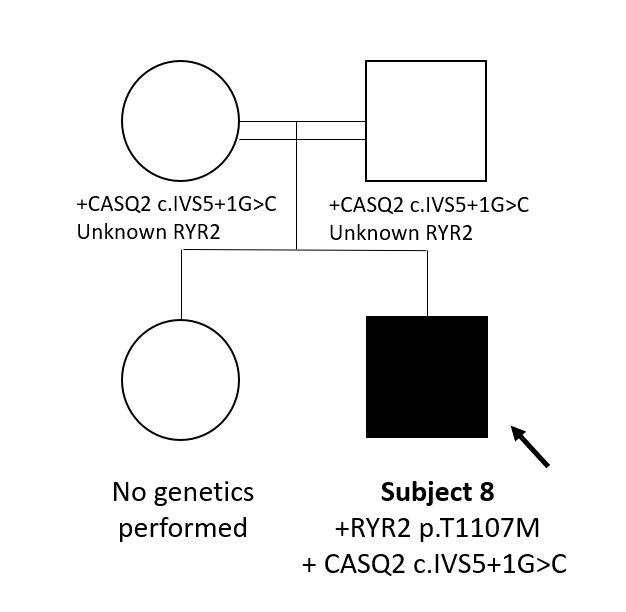


Pedigree 4: Family of Subject 8 (note parents are 1^st^ degree cousins)
